# The HDAC6/8/10 inhibitor TH34 induces DNA damage-mediated cell death in human high-grade neuroblastoma cell lines

**DOI:** 10.1007/s00204-018-2234-8

**Published:** 2018-06-09

**Authors:** Fiona R. Kolbinger, Emily Koeneke, Johannes Ridinger, Tino Heimburg, Michael Müller, Theresa Bayer, Wolfgang Sippl, Manfred Jung, Nikolas Gunkel, Aubry K. Miller, Frank Westermann, Olaf Witt, Ina Oehme

**Affiliations:** 1 Preclinical Program, Hopp Children’s Cancer Center at NCT Heidelberg (KiTZ), 69120 Heidelberg, Germany; 2 Clinical Cooperation Unit Pediatric Oncology, German Cancer Research Center (DKFZ), and German Cancer Consortium (DKTK), Im Neuenheimer Feld 280, 69120 Heidelberg, Germany; 3 Faculty of Biosciences, Heidelberg University, Im Neuenheimer Feld 234, 69120 Heidelberg, Germany; 4 Department of Medicinal Chemistry, Institute of Pharmacy, Martin-Luther-University Halle-Wittenberg, W.-Langenbeck-Str. 4, 06120 Halle, Germany; 5 Institute of Pharmaceutical Sciences, Albert-Ludwigs-Universität Freiburg, Albertstraße 25, 79104 Freiburg, Germany; 6 Cancer Drug Development Group, German Cancer Research Center (DKFZ), Im Neuenheimer Feld 580, 69120 Heidelberg, Germany; 7 Research Group Neuroblastoma Genomics, German Cancer Research Center (DKFZ), Im Neuenheimer Feld 280, 69120 Heidelberg, Germany; 8 Department of Pediatric Oncology, Hematology and Immunology, University Hospital Heidelberg, Im Neuenheimer Feld 430, 69120 Heidelberg, Germany

**Keywords:** Selective histone deacetylase inhibitor, HDAC8, HDAC10, DNA repair, Differentiation, Targeted therapy

## Abstract

High histone deacetylase (HDAC) 8 and HDAC10 expression levels have been identified as predictors of exceptionally poor outcomes in neuroblastoma, the most common extracranial solid tumor in childhood. HDAC8 inhibition synergizes with retinoic acid treatment to induce neuroblast maturation in vitro and to inhibit neuroblastoma xenograft growth in vivo. HDAC10 inhibition increases intracellular accumulation of chemotherapeutics through interference with lysosomal homeostasis, ultimately leading to cell death in cultured neuroblastoma cells. So far, no HDAC inhibitor covering HDAC8 and HDAC10 at micromolar concentrations without inhibiting HDACs 1, 2 and 3 has been described. Here, we introduce TH34 (3-(*N*-benzylamino)-4-methylbenzhydroxamic acid), a novel HDAC6/8/10 inhibitor for neuroblastoma therapy. TH34 is well-tolerated by non-transformed human skin fibroblasts at concentrations up to 25 µM and modestly impairs colony growth in medulloblastoma cell lines, but specifically induces caspase-dependent programmed cell death in a concentration-dependent manner in several human neuroblastoma cell lines. In addition to the induction of DNA double-strand breaks, HDAC6/8/10 inhibition also leads to mitotic aberrations and cell-cycle arrest. Neuroblastoma cells display elevated levels of neuronal differentiation markers, mirrored by formation of neurite-like outgrowths under maintained TH34 treatment. Eventually, after long-term treatment, all neuroblastoma cells undergo cell death. The combination of TH34 with plasma-achievable concentrations of retinoic acid, a drug applied in neuroblastoma therapy, synergistically inhibits colony growth (combination index (CI) < 0.1 for 10 µM of each). In summary, our study supports using selective HDAC inhibitors as targeted antineoplastic agents and underlines the therapeutic potential of selective HDAC6/8/10 inhibition in high-grade neuroblastoma.

**Electronic supplementary material:**

The online version of this article (10.1007/s00204-018-2234-8) contains supplementary material, which is available to authorized users.

## Introduction

Neuroblastoma is the most common extracranial solid tumor in childhood and the most frequently occurring cancer in infancy, accounting for 15% of pediatric cancer mortality (Brodeur 2003; Ward et al. 2014). Its clinical presentation is diverse, including highly differentiated local tumors with an excellent prognosis, spontaneously regressing metastatic disease and chemotherapy-resistant, invasive masses, which are likely to relapse. Treatment regimens for high-risk tumors involve dose-intensive chemotherapy, surgical resection and a combination of immunotherapy, antibodies and 13-*cis* retinoic acid (Cheung and Dyer 2013; Pinto et al. 2015; PDQ Pediatric Treatment Editorial Board, PDQ Cancer Information Summaries [Internet]. Bethesda (MD): National Cancer Institute (US) 2002–2017). Despite high-intensity chemotherapy, overall survival in high-risk neuroblastoma remains poor and chemotherapy-related toxicities are commonly observed. Thus, research has recently focused on the identification of novel, druggable targets and developing respective antineoplastic agents to abolish therapy resistance mechanisms and minimize chemotherapy-related adverse events.

The classical histone deacetylase (HDAC) family comprises 11 enzymatic subtypes, which, according to evolutionarily preserved catalytic domains, are divided into classes I (HDACs 1, 2, 3 and 8), IIa (HDACs 4, 5, 7 and 9), IIb (HDACs 6 and 10) and IV (HDAC11). Since HDACs catalyze the removal of acetyl groups from lysine residues of nuclear as well as cytoplasmic substrates, they affect diverse cellular processes including cell cycle control, apoptosis, metabolic homeostasis, stress response and autophagy (de Ruijter et al. 2003; Kim et al. 2001; Li and Zhu 2014; Yang and Seto 2008). Moreover, HDAC functions are protective against DNA damage, and depletion or inhibition of HDACs impair DNA damage repair mechanisms, rendering cells more susceptible to DNA-damaging agents (Miller et al. 2010). Recent evidence illustrates that HDAC inhibitors themselves propel DNA damage through replicative stress and a reduction of DNA repair proteins (Nikolova et al. 2017). HDACs are validated targets in anti-tumoral therapy and, to date, five HDAC inhibitors (panobinostat, romidepsin, belinostat, vorinostat and chidamide) have been approved for the treatment of hematological malignancies (Bates et al. 2015; Cheng et al. 2015; Mann et al. 2007; O’Connor et al. 2015; Shi et al. 2015). The approved HDAC inhibitors target multiple HDACs, including HDACs 1, 2 and 3, which are associated with serious, dose limiting adverse effects including leukopenia, thrombocytopenia, anorexia, vomiting, diarrhea and fatigue, mainly ascribed to an inhibition of HDACs 1, 2 and 3 (Bradner et al. 2010; Lane and Chabner 2009; Oehme et al. 2009a; Witt et al. 2009b). Selective targeting of tumor-relevant HDAC subtypes while avoiding inhibition of HDACs 1, 2 and 3 may thus lead to an increased therapeutic window with limited toxicity to healthy tissue (Balasubramanian et al. 2009).

HDAC8 is the only HDAC that is significantly transcriptionally upregulated in high-grade (INSS stage 4) neuroblastoma patient samples as compared to prognostically favorable stage 1, 2, 3 and 4S tumors. High HDAC8 expression strongly correlates with markers of poor prognosis (Oehme et al. 2009b). Selective HDAC8 inhibition induces a differentiated phenotype in neuroblastoma and reduces neuroblastoma growth in vitro and in vivo at least as effectively as unspecific HDAC inhibition while displaying fewer adverse effects (Rettig et al. 2015). This endorses selective HDAC8 inhibition as a very promising therapeutic option in neuroblastoma.

Class IIb HDACs 6 and 10 play an important role in protein degradation, lysosomal trafficking and cellular stress response (Kawaguchi et al. 2003; Koeneke et al. 2015; Kramer et al. 2014; Park et al. 2008; Yang and Seto 2008). HDAC10 expression strongly correlates with poor overall survival in high-grade (INSS stage 4) neuroblastoma (Oehme et al. 2013), making this HDAC a particularly attractive druggable target in this entity. HDAC10 supports neuroblastoma cell survival by promoting autophagic flux, and inhibition of HDAC10 sensitizes chemotherapy-resistant cells to treatment with DNA damage-inducing drugs such as doxorubicin (Oehme et al. 2013). In addition, HDAC10 promotes DNA damage repair (Radhakrishnan et al. 2015). HDAC6 expression does not significantly correlate with prognostic markers in neuroblastoma (Oehme et al. 2009b). HDAC6 and 10 share highly-conserved catalytic domains (Fischer et al. 2002) and structurally, it is therefore, utterly challenging to strictly avoid inhibition of one class IIb HDAC while significantly impairing the other subtype’s function. Inhibition of HDAC6 has been found to be well tolerated in preclinical and clinical studies (Santo et al. 2012; Vogl et al. 2017; Yee et al. 2016), which is why inhibition of HDAC6 did not lead to exclusion of a candidate inhibitor in this study.

Here, we present the novel small-molecule HDAC inhibitor TH34, which is the first HDAC inhibitor that shows pronounced selectivity for HDACs 6, 8 and 10 over HDACs 1, 2 and 3. Consistent with previous findings, treatment of neuroblastoma cells with TH34 induces signs of neuronal differentiation. Furthermore, we characterize DNA damage-inducing and cytotoxic effects of TH34 treatment in neuroblastoma, and identify the combination of the novel HDAC inhibitor with retinoic acid as synergistic and very effective in specifically eliminating tumor cells but not non-malignant fibroblasts. Taken together, our findings underline the specific roles of HDACs 8 and 10 in high-grade neuroblastoma and provide a rationale for further development of TH34 and all-*trans* retinoic acid (ATRA) as a treatment combination in this pediatric cancer entity.

## Materials and methods

### Cell culture

Human neuroblastoma cell lines SK-N-BE(2)-C (European Collection of Authenticated Cell Cultures, ECACC, Salisbury, UK), IMR-32 (German Collection of Microorganisms and Cell Cultures, DSMZ, Darmstadt, Germany), SK-N-AS (kindly provided by M. Schwab, DKFZ) and SH-SY5Y (DSMZ) as well as human medulloblastoma cell line MED8A (kindly provided by R. Gilbertson, St Jude Children’s Research Hospital, Memphis, TN, USA) and the non-transformed human foreskin fibroblast cell line VH7 (kindly provided by P. Boukamp, DKFZ) were cultured in Dulbecco’s Modified Eagle Medium (DMEM, Lonza, Basel, Switzerland) supplemented with 10% fetal calf serum (FCS, Sigma-Aldrich, Munich, Germany) and 1% non-essential amino acids (NEAA, Lonza). Kelly (DSMZ) and HD-MB03 (kindly provided by T. Milde, DKFZ) cells were cultured in RPMI 1640 medium (ThermoFisher Scientific, Braunschweig, Germany) containing 10% FCS and 1% NEAA. All cell lines were routinely authenticated using DNA fingerprinting authentication (DSMZ) and screened for mycoplasma contamination (Multiplexion, Heidelberg, Germany). All cell lines were cultured under standard conditions at 37 °C in a humidified atmosphere containing 5% CO_2_ and passages 15–30 were used.

### Primary neuroblastoma culture

Collection and use of neuroblastoma specimens was approved by the Institutional Review Board of the Medical Faculty, University of Heidelberg, and informed consent was obtained by the patient’s guardians. Bone marrow aspirates with high tumor cell infiltration were used to establish a primary culture. Briefly, neuroblastoma cells were isolated using a Ficoll gradient separation and subsequently cultured on matrigel-coated cell culture dishes in RPMI-1640 + 10% FCS for 7 days. Neuroblastoma spheroids were then sub-cultured for 2–3 passages before freezing in 10% DMSO + 20% FCS.

### Cell culture reagents and chemicals

TH34 (3-(*N*-benzylamino)-4-methylbenzhydroxamic acid) (stock concentration 50 mM) was synthesized by coauthors TH and WS as described previously (Heimburg et al. 2016, 2017) and was dissolved in DMSO (Sigma-Aldrich). All*-trans* retinoic acid (ATRA, Sigma-Aldrich, stock concentration 10 mM) was dissolved in ethanol (EtOH, Sigma-Aldrich). Z-VAD-FMK (Biozol, Eching, Germany, stock concentration 100 mM), necrostatin-1 (Cayman Chemical, Tallinn, Estonia, stock concentration 50 mM) and trolox (kindly provided by N. Brady, Johns Hopkins Bloomberg School of Public Health, Baltimore, MD, USA, stock concentration 50 mM) were dissolved in DMSO. *N*-Acetylcysteine (NAC, Sigma-Aldrich, stock concentration 1 mM) was dissolved in autoclaved Millipore H_2_O and stored at 4 °C protected from light. If not otherwise specified, compounds were stored at − 20 °C and protected from light.

### NanoBRET assay

HeLa cells, stably transfected with NanoBRET plasmids NanoLuc^®^-HDAC6 FL Fusion Vector and NanoLuc^®^-HDAC10 FL Fusion Vector (Promega, Madison, WI, USA) were seeded at 20,000 cells/well in white 96-well plates. Without further incubation, tracer (0.3 µM) and drugs were added in separate steps and plates were placed in a tissue culture incubator for 2 h. For NanoBRET quantification, plates were put at room temperature for 10 min. Nanoglow substrate, diluted in OptiMEM without phenol red, was added and measured within 10 min in an OPTIMA plate reader (460 nm emission for donor and 610LP filter for acceptor, BMG Labtech, Ortenberg, Germany). The BRET signal was calculated by the ratio of acceptor signal to donor signal.

### Western blot analysis

Western blot analysis was performed as described previously described (Oehme et al. 2009b). The following antibodies were used: anti-histone 3 (#9715, Cell Signaling Technology, Leiden, The Netherlands), anti-acetylated histone 3 (#06-911, Millipore), anti-tubulin (#2148, Cell Signaling Technology), anti-acetylated tubulin (#6793, Sigma-Aldrich), anti-acetylated SMC3 (kindly provided by Katsuhiko Shirahige, Institute for Molecular and Cellular Biosciences, University of Tokyo, Japan (Nishiyama et al. 2010)), anti-HSC70/HSP70 (#sc-33575, Santa Cruz Biotechnology, Heidelberg, Germany), and anti-β-actin (#5441, Sigma-Aldrich).

### Acridine orange staining

Acridine orange-positive acidic vesicular organelles were detected as previously described (Oehme et al. 2013).

### Determination of biochemical HDAC3 activity

Ten doses of TH34 were tested by the company Reaction Biology Corp. (Malvern, PA, USA) against a specific fluorogenic HDAC3 substrate (peptide RHKK(Ac)AMC from p53 residues 379–382) in three-fold serial dilution, starting at 1000 µM. Trichostatin A (threefold serial dilution starting at 10 µM) served as a positive control and IC50 values were calculated using GraphPad Prism version 5.01 (GraphPad Software).

### Class IIa HDAC activity assay

Class IIa HDAC activity was performed as previously described (Ecker et al. 2015).

### Cell viability analysis

Adherent cells were detached using trypsin–EDTA (ThermoFisher Scientific) and pooled with corresponding supernatant, centrifuged and resuspended in 1 ml of cell culture medium. Cell viability was measured by automated trypan blue staining using the Vi-Cell XR Cell Viability Analyzer (Beckman Coulter, Krefeld, Germany). Caspase-3-like protease activity was analyzed as previously described (Oehme et al. 2009b).

### Cell cycle analysis

Cell cycle analysis was performed as previously described (Oehme et al. 2006).

### Histone protein H2AX phosphorylation assay (γH2AX)

Phosphorylation of histone protein H2AX on serine 139 indicates DNA double-strand breaks and blockage of replication forks (Mariotti et al. 2013; Muslimovic et al. 2008). After treatment as indicated, 4 × 10^5^ viable cells were transferred to a round-bottom plate. Cells were fixed and permeabilized using buffers using the eBioscience™ Foxp3/Transcription Factor Staining Buffer Set (ThermoFisher Scientific) according to manufacturer’s protocol. Subsequently, cells were incubated for 1.5 h with γH2AX primary antibody (ThermoFisher Scientific) on ice, washed twice and incubated with anti-rabbit Alexa Fluor^®^ 488 secondary antibody for 1 h on ice before washing and measurement utilizing a FACSCanto II Flow Cytometer (Becton, Dickinson and Company).

### Fluorescence microscopic analysis of NEFM and γH2AX staining

SK-N-BE(2)-C cells were seeded at a density of 2 × 10^4^ cells per well into an ibidi 8-well µ-slide and treated for 24 h as indicated. Adherent cells were washed with PBS, fixed for 15 min using 4% paraformaldehyde (PFA) at room temperature, permeabilized for 30 min with 0.2% Triton-X 100 (ThermoFisher Scientific) in phosphate-buffered saline (PBS) and blocked with 3% BSA in 0.05% Triton-X 100 (ThermoFisher Scientific) in PBS for 1 h at room temperature. Primary antibodies were diluted 1:500 (NEFM, Millipore) or 1:200 (γH2AX, Cell Signaling Technology) and after overnight incubation, cells were incubated with fluorescent secondary antibody for 2 h at room temperature and counterstained with DAPI. Images were acquired on a CKX41 light microscope (Olympus, Hamburg, Germany) with a reflected fluorescence system or a Zeiss LSM710 laser scanning confocal microscope (Carl Zeiss, Oberkochen, Germany).

### Quantitative real-time PCR

Real-time PCR was performed as described previously (Fischer et al. 2005; Witt et al. 2003). Unless otherwise indicated, primers were purchased from ThermoFisher Scientific, and the following primers were used: *CDKN1A* (p21^WAF1/CIP1^, forward: 5′-TGGAGACTCTCAGGGTCGAAA-3′, reverse: 5′-GGCGTTTGGAGTGGTAGAAATC-3′), *HPRT* (forward: 5′-TGACACTGGCAAAACAATGCA-3′, reverse: 5′-GGTCCTTTTCACCAGCAAGCT-3′), *NTRK1* (forward: 5′-CAGCCGGCACCGTCTCT-3′, reverse: 5′-TCCAGGAACTCAGTGAAGATGAAG-3′), *PUMA* (forward: 5′-CCTGGAGGGTCCTGTACAATCT-3′, reverse: 5′-GCACCTAATTGGGCTCCATCT-3′), *SDHA* (forward: 5′-TGGGAACAAGAGGGCATCTG-3′, reverse: 5′-CCACCACTGCATCAAATTCATG-3′). Data are expressed as relative gene expression (fold change) according to the 2^−ΔΔCt^ method (Livak and Schmittgen 2001), normalized to neuroblastoma housekeeping genes SDHA and HPRT (Fischer et al. 2005) and set in relation to negative control.

### Cell differentiation assay

Adherent cells plated on 6-well plates were treated as indicated. For staining, cells were rinsed once with (PBS) and incubated with crystal violet staining solution (1% (w/v) in 70% EtOH) for 1 min. Subsequently, the staining solution was removed and cells were rinsed two to three times with autoclaved purified water and allowed to dry. A semi-automated macro determining surface of cell bodies as well as number and length of neurites was used for evaluation of ten fields of vision in ImageJ version 1.49v and normalized to solvent control.

### Colony formation assay

Cells were plated on 6-well plates at a density of 500 (SK-N-BE(2)-C), 750 (MED8A) or 1,000 (IMR-32, Kelly, SH-SY5Y, SK-N-AS, HD-MB03) cells per well and treated as indicated for 96 h. Adherent cells were washed three times with PBS and cultured for 7 additional days before staining of viable cell colonies with crystal violet and quantification using ImageJ version 1.49v (Schneider et al. 2012). For synergism calculation, combination indices were determined from quantified colony growth using the CompuSyn synergism calculation software based on the Chou–Talalay method (Chou 2010).

### CellTiter-Glo assay

Cells were plated on 96-well plates at a density of 10,000 (SK-N-BE(2)-C, IMR-32, Kelly) or 20,000 (NB8) cells per well and treated as indicated for 72 h. According to manufacturer’s protocol, cells were incubated with reagent for 25 min using the CellTiter-Glo 2.0 kit (Promega, SK-N-BE(2)-C, IMR-32, Kelly) or the CellTiter-Glo 3D kit (Promega, NB8) and bioluminescence was read in an OPTIMA plate reader (BMG Labtech).

### Statistical analysis

Data are presented as mean ± standard deviation (SD). All cell culture experiments were performed in duplicate or triplicate, and each experiment was repeated at least three times. A two-tailed unpaired *t* test was performed using GraphPad Prism version 5.01 (GraphPad Software) to compare treatment groups, and p values of less than 0.05 were considered to be significant (*p < 0.05, **p < 0.01, ***p < 0.001).

## Results

### The HDAC inhibitor TH34 selectively inhibits HDACs 6, 8 and 10

**Fig. 1:**
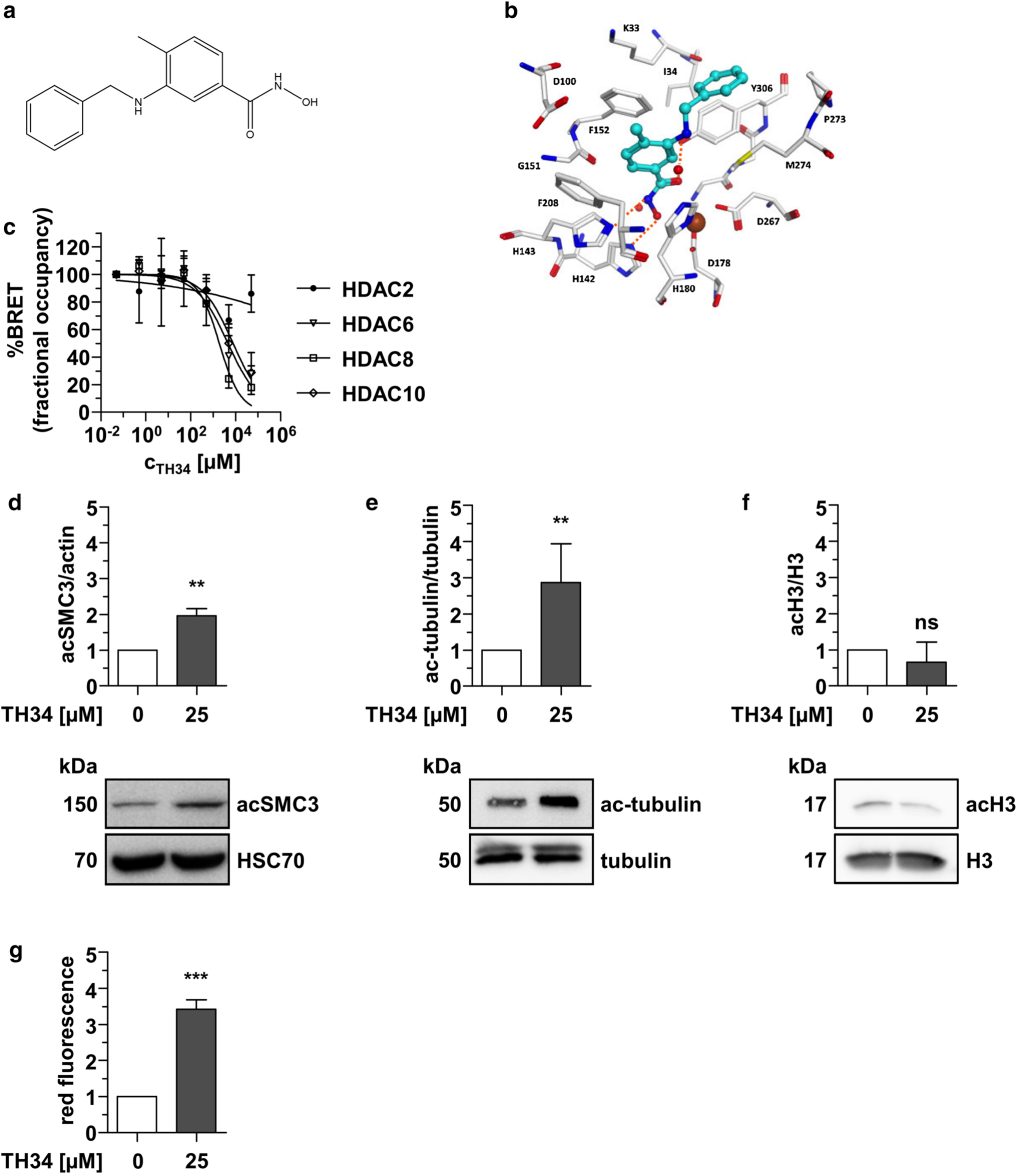
TH34 inhibits HDACs 6, 8 and 10. **a** TH34 molecular structure. **b** Docking pose of TH34 (middle, cyan color) at HDAC8. **c** NanoBRET analysis of HDAC2/6/8/10 interaction with TH34 in HeLa cells. The number of biological replicates is *n* = 3 for HDAC6 and HDAC10 and *n* = 2 for HDAC2 and HDAC8 and the number of technical replicates is *n* = 3 for every independent run. Graph represents mean amounts of acceptor-occupied NanoLuc-HDAC2/6/8/10 relative to the total amount of NanoLuc-HDAC2/6/8/10 (% fractional occupancy, *y*-axis) versus logarithmic drug concentration (*x*-axis). Western Blot analysis of SMC3 (**d**), tubulin (**e**) and histone 3 acetylation (**f**) in SK-N-BE(2)-C cells after 6 h of treatment with TH34 (25 µM) or solvent. **g** Flow-cytometric quantification of acridine orange-positive acidic vesicular organelles in SK-N-BE(2)-C neuroblastoma cells after 24 h of treatment. Bar graphs represent mean values of at least three independent experiments performed in triplicates and statistical analysis was performed using unpaired, two-tailed *t* test (***p < 0.001; **0.001 ≤  *p* < 0.01; *0.01 ≤  *p* < 0.05, *ns* not significant). Error bars represent SD

As no such compound has been developed to date, we sought to develop a novel HDAC inhibitor with high selectivity for HDAC8 and HDAC10 over HDACs 1, 2 and 3, and tolerated HDAC6 inhibition during the screening process (Fig. 1a, b). Following computer-based screening of an in-house library of hydroxamic acids, inhibitors displaying promising effects in cell-free biochemical assays were tested for specificity and anti-tumor effects in multiple pediatric cancer cell lines, including high-grade neuroblastoma cells (Heimburg et al. 2017). Fluorometric determination of biochemical HDAC inhibition displayed no substantial effect against HDACs 1, 3 (Supplementary Table, Supplementary Fig. 1a, (Heimburg et al. 2017)) as well as class IIa HDACs (Supplementary Table, Supplementary Fig. 1b, c). Measurement of cellular target engagement with a NanoBRET assay (Robers et al. 2015) revealed that candidate inhibitor TH34 (Fig. 1a, b) strongly binds HDAC6, 8 and 10 with low-micromolar IC_50_ concentrations (HDAC6: 4.6 µM, HDAC8: 1.9 µM, HDAC10: 7.7 µM), and shows no substantial affinity to HDAC2 at concentrations up to 50 µM (Fig. 1c). Analysis of intracellular substrate acetylation in SK-N-BE(2)-C high-grade neuroblastoma cells confirmed these findings. Whereas acetylation of the specific HDAC8 target structural maintenance of chromosomes protein 3 (SMC3, Fig. 1d) and the HDAC6 target tubulin (Fig. 1e) significantly increased after 6 h of treatment with TH34, histone 3 (H3) acetylation status remained unchanged (Fig. 1f), indicating no effect on HDACs 1, 2 and 3. Moreover, treatment of SK-N-BE(2)-C cells with TH34 induced strong accumulation of acidic vesicles, quantified via flow-cytometric analysis using the acidophilic dye acridine orange (Fig. 1g). This cellular phenotype is observed after specific knockdown or inhibition of HDAC10 (Oehme et al. 2013) and was used as a method to further support intracellular HDAC10 inhibition.

### TH34 induces caspase-dependent programmed cell death in neuroblastoma cells

**Fig. 2:**
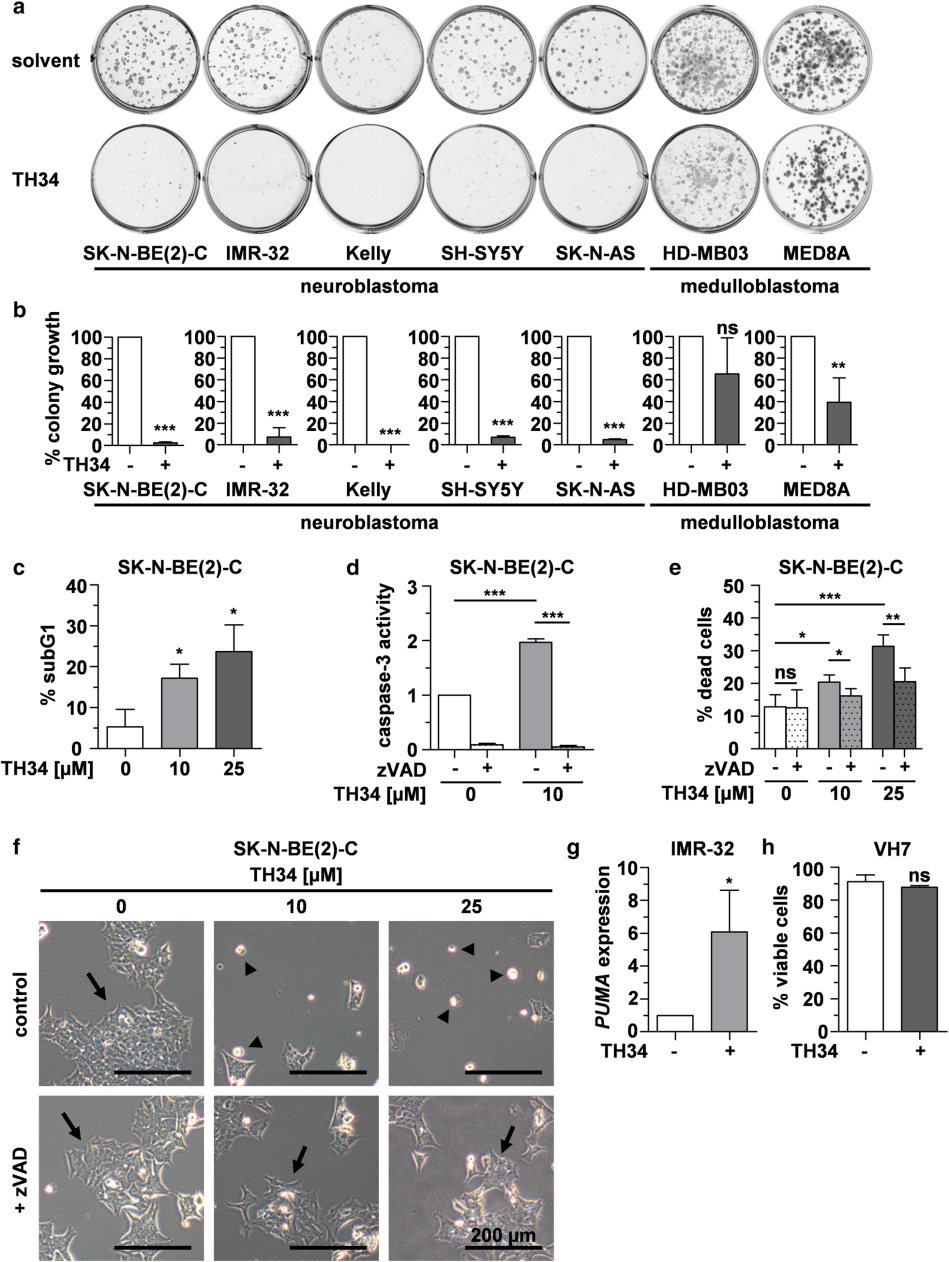
TH34 induces caspase-dependent programmed cell death in neuroblastoma cells. **a, b** Colony growth after treatment with TH34 (25 µM) or solvent. Representative images and quantification of colony growth in at least three independent experiments performed in triplicates are shown for each cell line. **c** Fraction of cells in subG1 cell cycle phase after treatment with indicated concentrations of TH34 for 72 h, identified via flow-cytometric quantification of DNA content using propidium iodide. **d** Caspase-3 activity after treatment of SK-N-BE(2)-C cells with indicated concentrations of TH34 for 48 h with or without Z-VAD-FMK (20 µM). **e** Proportion of dead SK-N-BE(2)-C cells after treatment with different concentrations of TH34 for 72 h with or without Z-VAD-FMK (20 µM), determined via automated trypan blue staining. **f** Representative images of SK-N-BE(2)-C neuroblastoma cells treated with solvent or TH34 (25 µM) with or without Z-VAD-FMK (20 µM) for 72 h. **g** Relative expression (determined using the 2^−ΔΔCt^ method and normalized to solvent control) of *PUMA* in IMR-32 cells after 24 h of treatment with TH34 (10 µM). **(h)** VH7 non-malignant fibroblast viability after treatment with solvent or TH34 (25 µM) for 72 h. Bar graphs represent mean values of at least three independent experiments performed in triplicates and statistical analysis was performed using unpaired, two-tailed *t* test (***p < 0.001; **0.001 ≤  *p* < 0.01; *0.01 ≤  *p* < 0.05, *ns* not significant). Error bars represent SD

To investigate the long-term effect of a simultaneous inhibition of HDAC6, 8 and 10 on pediatric cancer cell lines, we treated five neuroblastoma and two medulloblastoma cell lines, featuring different genetic aberrations, with TH34 for 4 days and allowed remaining cells to regrow colonies in fresh media for another 7 days. TH34 treatment abolished colony growth in neuroblastoma cells independent of their genetic status, whereas the effect was not as pronounced in medulloblastoma cell lines (Fig. 2a, b).

To characterize tumor cell death induced by combined inhibition of HDAC6, 8 and 10, we quantified the fraction of cells in subG1 cell cycle phase after 72 h of TH34 treatment. TH34 significantly increased the subG1 fraction of SK-N-BE(2)-C cells in a dose-dependent manner (Fig. 2c). We then treated and co-incubated cells with TH34 and the pan-caspase inhibitor Z-VAD-FMK. TH34 activated effector caspases and cell death in SK-N-BE(2)-C high-grade neuroblastoma cells in a concentration-dependent manner, which could be rescued by addition of Z-VAD-FMK (Fig. 2d–f). At the same time, TH34-induced cell death could not be significantly rescued by addition of potent inhibitors of ROS-dependent cell death (*N*-acetylcysteine, NAC, Supplementary Fig. 2a), necroptosis (necrostatin-1, Supplementary Fig. 2b) and oxidative stress-induced cell death (trolox, Supplementary Fig. 2c). Furthermore, expression of pro-apoptotic p53 upregulated modulator of apoptosis (*PUMA*) was significantly increased in IMR-32 (*TP53* wild-type) cells following 72 h of TH34 treatment (Fig. 2g). In *TP53*-mutated SK-N-BE(2)-C cells, *PUMA* expression could not be detected.

To control for cytotoxic effects of TH34 on healthy cells, we treated proliferating non-malignant fibroblasts (VH7 cells) with this compound for 72 h. In contrast to our findings in SK-N-BE(2)-C cells, TH34 exhibited very limited cytotoxic effects in fibroblasts (Fig. 2h). Taken together, these results indicate that cell death resulting from simultaneous HDAC6, 8 and 10 inhibition is mainly a caspase-dependent programmed type of cell death, such as apoptosis.

**Fig. 3:**
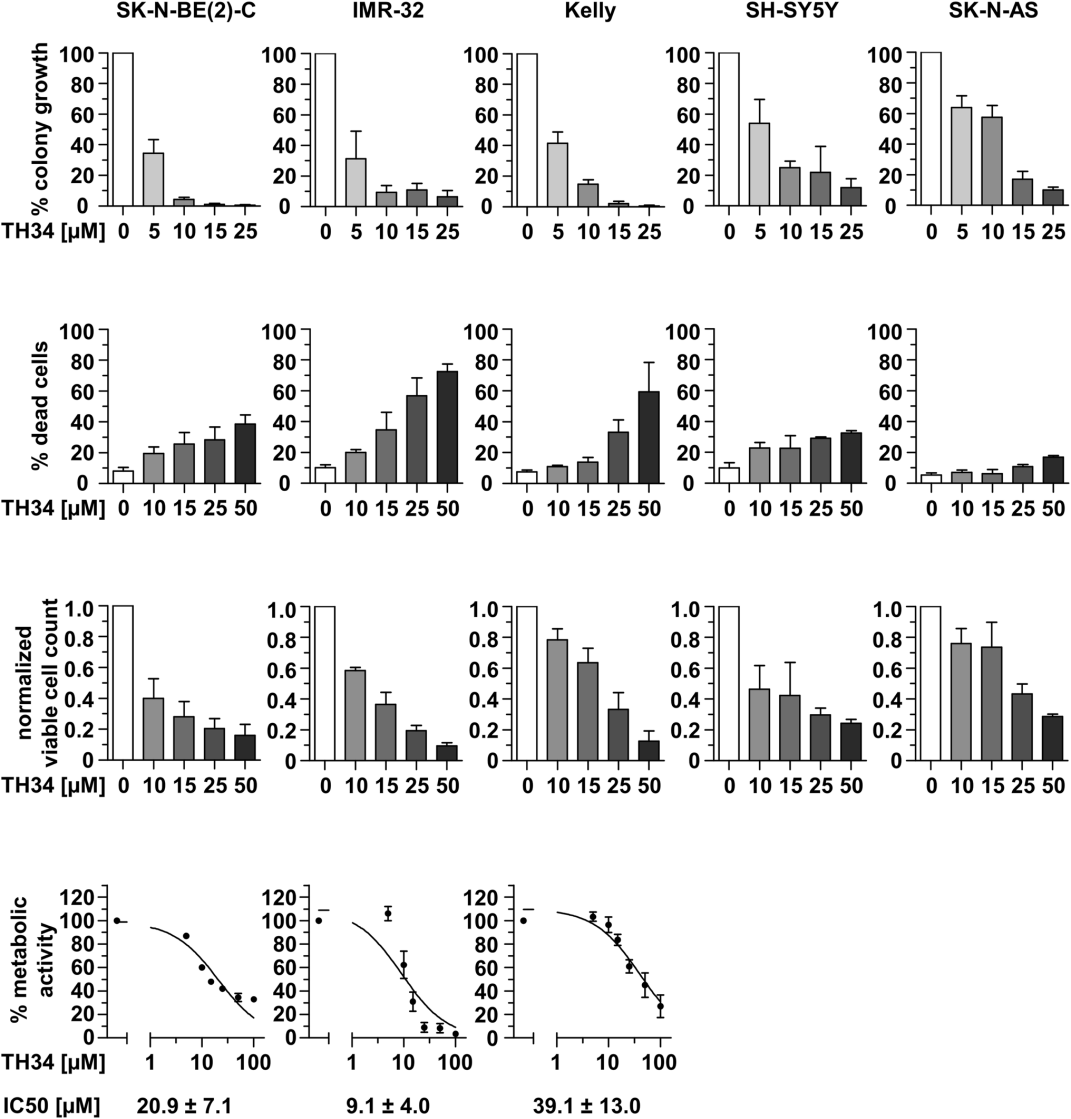
TH34 differentially impairs colony formation and cell survival in neuroblastoma cell lines with distinct molecular features. Relative colony formation, proportion of dead cells and viable cell count (both determined via trypan blue exclusion assay) as well as metabolic activity (CellTiter-Glo) in five different neuroblastoma cell lines (SK-N-BE(2)-C, IMR-32, Kelly, SH-SY5Y and SK-N-AS) after treatment with indicated concentrations of TH34. Bar graphs represent mean values of at least two independent experiments performed in triplicates each and error bars represent SD

To further compare the response of neuroblastoma cell lines to TH34 treatment, we investigated colony formation, cell viability, viable cell count and cellular metabolic activity in five neuroblastoma cell lines after treatment with increasing doses of TH34 (Fig. 3). These results indicate nuances in the responsiveness of neuroblastoma cell lines to HDAC6/8/10 inhibition. *MYCN*-amplified cell lines (BE(2)-C, IMR-32, Kelly) tend to appear more sensitive to TH34 treatment than *MYCN* single-copy cell lines (SK-N-AS and SH-SY5Y). In addition, the *TP53* wildtype cell line IMR-32 was highly responsive in terms of cell death. Thus, although neuroblastoma cells are more sensitive to TH34 than cell lines of other tumor entities, even neuroblastoma cells show diverse responses, possibly related to different *MYCN* expression levels (amplification versus single copy).

### TH34 induces differentiation and cell cycle arrest in neuroblastoma cells

**Fig. 4:**
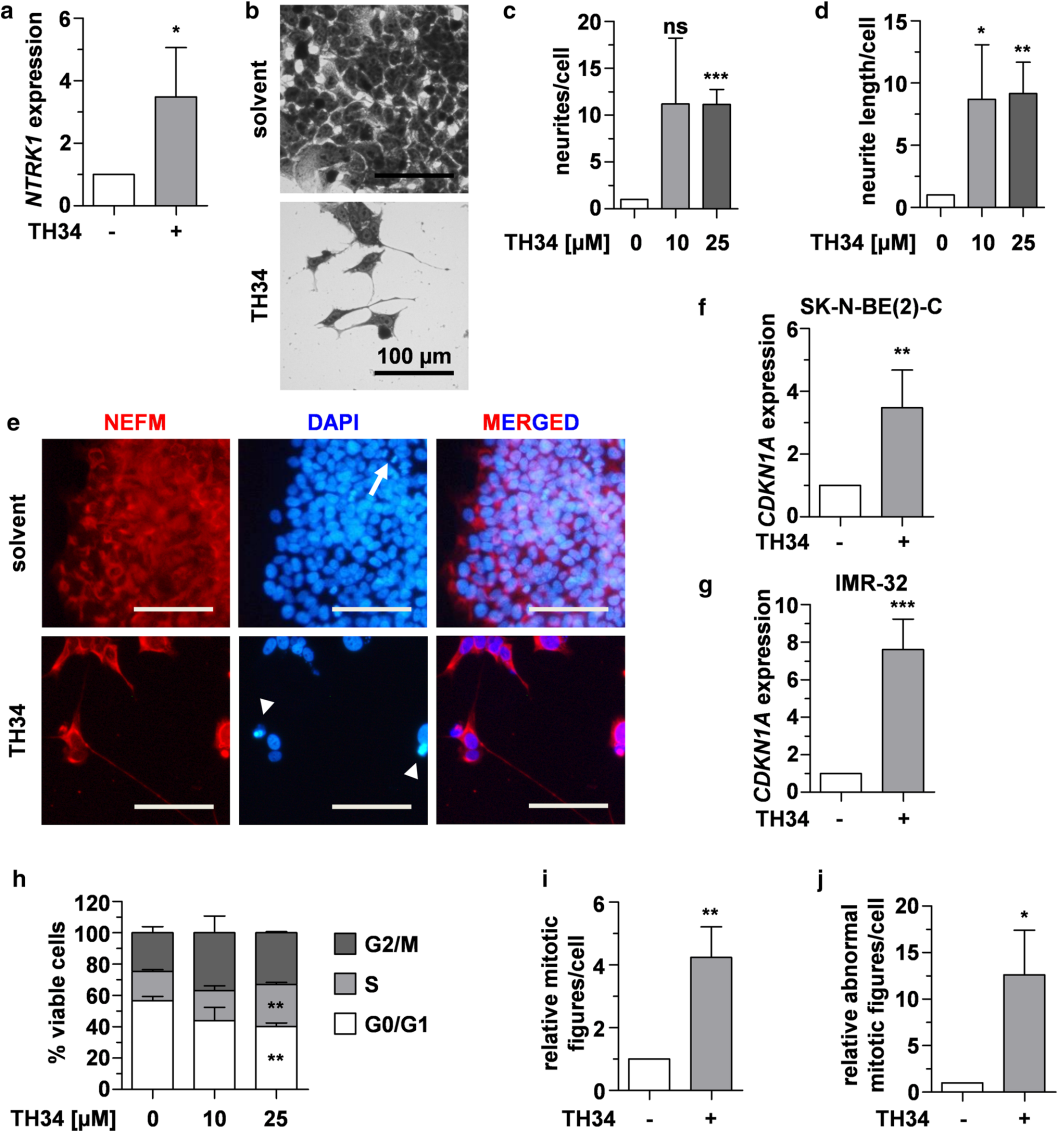
TH34 induces differentiation and cell cycle arrest in neuroblastoma cells. **a** Relative expression (determined using the 2^−ΔΔCt^ method and normalized to solvent control) of *NTRK1* in SK-N-BE(2)-C neuroblastoma cells after 72 h of treatment with TH34 (10 µM). **b** Representative microscopic images of crystal violet-stained SK-N-BE(2)-C cells treated with TH34 (10 µM) for 6 days. **c**–**d** Relative number and length of neurites in SK-N-BE(2)-C cells after 6 days of treatment with indicated concentrations of TH34, quantified using a semi-automated macro determining surface of cell bodies as well as number and length of neurites in ten fields of vision in ImageJ version 1.49v and normalized to solvent control. **e** Fluorescence microscopic analysis of neurofilament M expression in SK-N-BE(2)-C cells treated with TH34 (10 µM) for 6 days. Nuclei were counterstained with DAPI, arrows and arrowheads indicate normal mitotic and aberrant mitotic nuclei, respectively. Relative expression (determined using the 2^− ΔΔCt^ method and normalized to solvent control) of *CDKN1A* in SK-N-BE(2)-C **(f)** and IMR-32 **(g)** neuroblastoma cells after 72 h of treatment with TH34 (10 µM). **h** Cell cycle distribution of viable SK-N-BE(2)-C cells in G0/G1 (white), S (light gray) and G2/M (dark gray) phase after 72 h of treatment with indicated concentrations of TH34. **i, j** Total and aberrant mitotic nuclei in SK-N-BE(2)-C neuroblastoma cells after 6 days of treatment with TH34 (10 µM). In ten fields of vision, all DAPI-stained nuclei and mitotic figures were counted using the Cell Counter Plugin for ImageJ version 1.49v and numbers obtained in treated samples were set in relation to solvent control. Bar graphs represent mean values of at least three independent experiments and statistical analysis was performed using unpaired, two-tailed *t* test (***p < 0.001; **0.001 ≤ *p* < 0.01; *0.01 ≤ *p* < 0.05, *ns* not significant). Error bars represent SD

As neuroblastoma arises from immature neuroblasts, differentiation induction is a pivotal part of current therapeutic regimens. HDAC8 has been identified to play a role in maintenance of an undifferentiated cellular phenotype (Rettig et al. 2015). We thus investigated whether TH34 treatment would enhance expression of neuronal differentiation markers. After 72 h of treatment with TH34, expression levels of the neurotrophic receptor tyrosine kinase 1 (*NTRK1*, Fig. 4a) significantly increased. In neuroblastoma, *NTRK1* expression is associated with benign clinical features (e.g., young age, favorable pathology and non-*MYCN* amplified genetic status), tumor cell differentiation and good outcome (Pajtler et al. 2014; Schramm et al. 2012). After 6 days of treatment with TH34, SK-N-BE(2)-C neuroblastoma cells markedly altered their morphology, characterized by formation of neurite-like structures (Fig. 4b). Under TH34 treatment, the neurites not only significantly increased in number (Fig. 4c) but also in length (Fig. 4d). Further underlining the differentiating effect of HDAC6/8/10 inhibition, SK-N-BE(2)-C cells treated with TH34 showed strongly positive neurofilament M (NEFM) staining (Fig. 4e). Low-micromolar doses of TH34 enhanced ATRA-induced morphological features of differentiation in a similar manner as we had previously described with the selective HDAC8 inhibitor PCI-34051 (Rettig et al. 2015) (Supplementary Fig. 3a). This drug combination also synergistically reduced neuroblastoma colony growth (CI < 0.1 for 10 µM of each) at concentrations of ATRA that can be maintained in human plasma (Adamson 1996) (Supplementary Fig. 3b–d).

To further characterize the effects mediated by TH34, we examined the expression of the cell cycle inhibitor *CDKN1A* (p21^WAF1/CIP1^). In response to cellular stress conditions, *CDKN1A* expression is induced through p53-dependent and -independent pathways (El-Deiry et al. 1993; Gartel and Tyner 1999, reviewed by; Jung et al. 2010). We found dose-dependent upregulation of *CDKN1A* in both *TP53*-mutated (SK-N-BE(2)-C, Fig. 4f) and *TP53*-wild type neuroblastoma cells (IMR-32, Fig. 4g) after 72 h of TH34 treatment. Among living SK-N-BE(2)-C cells, we observed a marked shift from G0/G1 phase to S/G2/M phase after 72 h of TH34 treatment (Fig. 4h).

The protein SMC3 is a specific substrate for HDAC8 (Deardorff et al. 2012) and, together with structural maintenance of chromosomes 1A (SMC1), RAD21 and stromal antigen 1/2 (STAG1/2), SMC3 constitutes the cohesin complex, which forms a ring-like structure interconnecting sister chromatids during mitosis (Uhlmann 2016). SMC3 undergoes a cycle of deacetylation and acetylation, which is essential for cohesin functions (Beckouet et al. 2010). Although the impact of SMC3 hyperacetylation on mitosis and cell cycle progression remains to be fully elucidated, recent studies suggest that hyperacetylation inhibits sister chromatid release at anaphase (Beckouet et al. 2016; Gligoris et al. 2014). Since TH34 treatment induces hyperacetylation of SMC3 in high-grade neuroblastoma cells by blocking HDAC8 (Fig. 1d), we investigated if nuclear morphology and abundance of intact and aberrant mitotic figures changed under TH34 treatment. Indeed, 4′,6-diamidino-2-phenylindole (DAPI) staining revealed a significantly higher number of mitotic cells after treatment with HDAC inhibitor TH34 (Fig. 4e, i). Moreover, TH34-treated cells much more frequently displayed increased nuclear size and mitoses with aberrant features such as multipolar or asymmetric spindles than control cells (Fig. 4e, j and Supplementary Fig. 4). Taken together, our results indicate that concomitant inhibition of HDACs 6, 8 and 10 induces signs of neuron-like differentiation, cell cycle arrest and mitotic aberrations in neuroblastoma cells.

### TH34 induces DNA damage in high-grade neuroblastoma cells

Induction of DNA damage and inhibition of cellular DNA repair bear the potential to take advantage of malignant cells’ dysfunctional DNA damage response mechanisms and thus drive them into cell death while sparing non-malignant cells (Hosoya and Miyagawa 2014; Lord and Ashworth 2012). Treatment of cancer cells with broad-spectrum HDAC inhibitors such as vorinostat (suberoylanilide hydroxamic acid, SAHA) and trichostatin A (TSA) enhance DNA damage and impair non-homologous end joining (NHEJ) of DNA double-strand breaks (DSBs) (Lee et al. 2010; Pang et al. 2016; Robert et al. 2016; Vashishta and Hetman 2014). The exact role of single HDAC subtypes including HDAC8 and HDAC10 in DNA damage repair, however, is not yet fully understood.

**Fig. 5:**
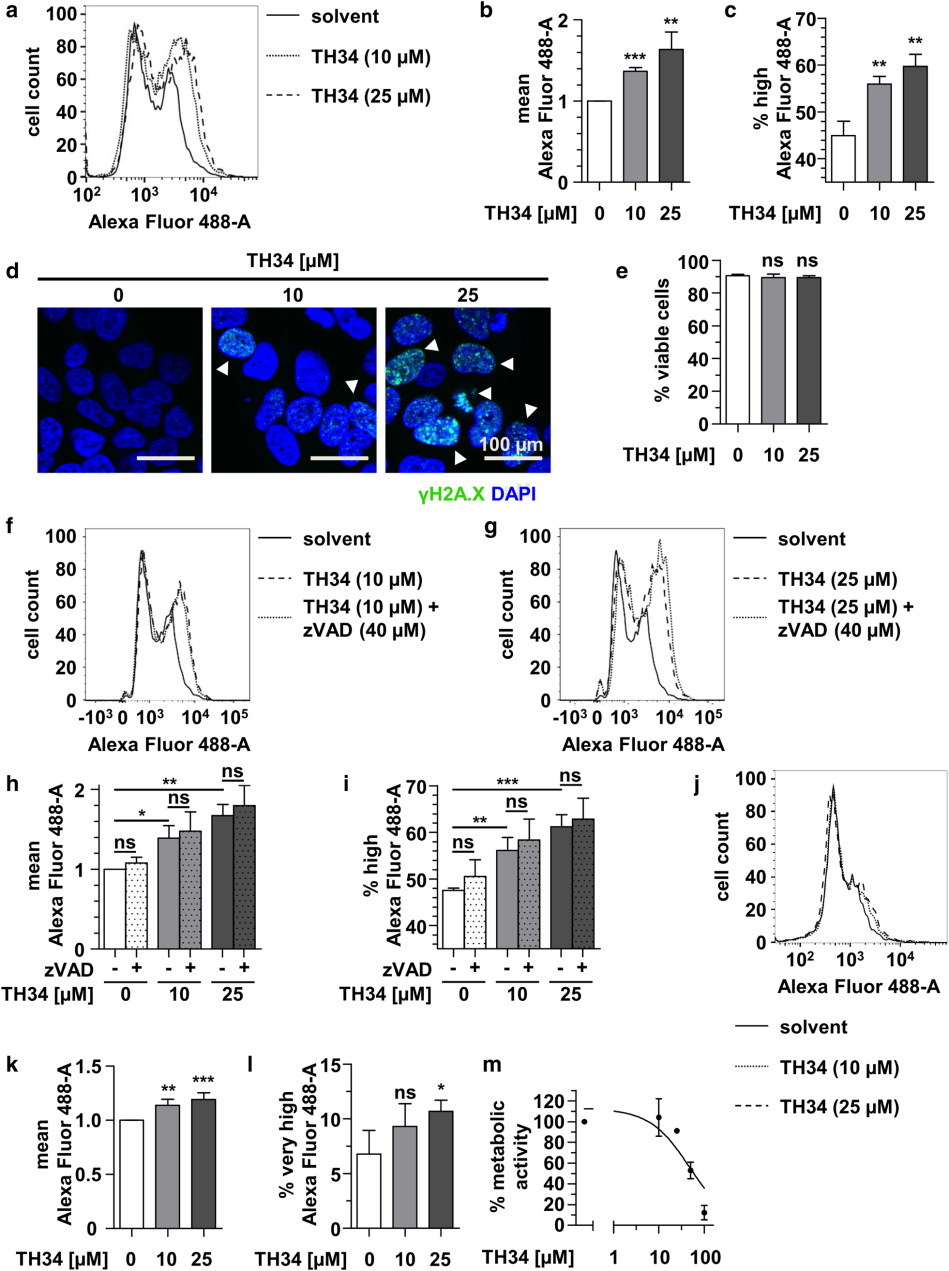
TH34 induces DNA damage in high-grade neuroblastoma cells. **a** Flow cytometric analysis of H2AX S134 phosphorylation (γH2AX) in fixed, viable SK-N-BE(2)-C cells after 24 h of treatment with indicated concentrations of TH34. Figure shows histogram of logarithmic fluorescence (*x*-axis) versus event number (*y*-axis) and gate represents γH2AX-positive cells. Mean Alexa Fluor 488 fluorescence (**b**) and proportion of γH2AX-positive cells (**c**) after treatment of SK-N-BE(2)-C cells with different concentrations of TH34 for 24 h. **d** Immunofluorescence analysis of γH2AX staining on fixed SK-N-BE(2)-C cells after 24 h of treatment with indicated concentrations of TH34. Nuclei were counterstained with DAPI, and arrowheads indicate nuclei with high number of DNA double-strand breaks. **e** Proportion of dead SK-N-BE(2)-C cells after treatment with different concentrations of TH34 for 24 h, determined via automated trypan blue staining. **f, g** Flow cytometric analysis of H2AX S134 phosphorylation (γH2AX) in fixed viable SK-N-BE(2)-C cells after 1 h of pre-treatment with Z-VAD-FMK (40 µM) alone and 24 h of additional treatment with TH34 at a concentration of 10 µM (**f**) or 25 µM (**g**). Figure shows histogram of logarithmic fluorescence (*x*-axis) versus event number (*y*-axis) and gate represents γH2AX--positive cells. Mean Alexa Fluor 488 fluorescence (**h**) and proportion of γH2AX-positive cells (**i**) after 1 h of Z-VAD-FMK (40 µM) pre-treatment of SK-N-BE(2)-C cells and additional treatment with different concentrations of TH34 for 24 h. **j** Flow cytometric analysis of H2AX S134 phosphorylation (γH2AX) in fixed viable NB8 primary neuroblastoma cells after 24 h of treatment with indicated concentrations of TH34. Figure shows histogram of logarithmic fluorescence (*x*-axis) versus event number (*y*-axis) and gate represents γH2AX-positive cells. Mean Alexa Fluor 488 fluorescence (**k**) and proportion of γH2AX highly positive cells (**l**) after treatment of NB8 primary neuroblastoma cells with different concentrations of TH34 for 24 h. **(m)** Cellular metabolic activity (CellTiter-Glo) of NB8 primary neuroblastoma cells after treatment with indicated concentrations of TH34 for 72 h. Bar graphs represent mean values of at least three independent experiments and statistical analysis was performed using unpaired, two-tailed *t* test (***p < 0.001; **0.001 ≤ *p* < 0.01; *0.01 ≤ *p* < 0.05, *ns* not significant). Error bars represent SD

Thus, we investigated whether mutual inhibition of HDACs 6, 8 and 10 affected DNA integrity. Flow-cytometric analysis of H2AX phospho-S134 (γH2AX) in viable SK-N-BE(2)-C cells revealed a dose-dependent significant increase in γH2AX-positive cells after treatment with TH34 for 24 h (Fig. 5a–c). Immunofluorescence staining of γH2AX indicated nuclear foci in TH34-, but not solvent-treated high-grade neuroblastoma cells, confirming dose-dependent occurrence of DSBs under HDAC6/8/10 inhibition (Fig. 5d). Of note, we did not observe relevantly decreased cell viability nor aberrant mitotic figures in SK-N-BE(2)-C cells after 24 h of treatment with TH34, meaning that DNA damage occurred prior to cell death of neuroblastoma cells (Fig. 5e).

As DNA double-strand breaks can result from caspase activation (Rogakou et al. 2000), we aimed to rule out unspecific γH2AX positivity due to caspase activation. Thus, we analyzed whether 1 h of Z-VAD-FMK pre-treatment of SK-N-BE(2)-C cells and additional treatment with different concentrations of TH34 for 24 h affects TH34-induced γH2AX positivity in SK-N-BE(2)-C cells. Again, treatment of cells with TH34 dose-dependently increased γH2AX-positivity, which was unaffected by Z-VAD-FMK co-treatment (Fig. 5f–i). Finally, we investigated the effect of a combined inhibition of HDAC6, 8 and 10 in short-term cultures of primary neuroblastoma cells isolated from a *MYCN*-amplified INSS stage 4 tumor of a 1-month-old patient (NB8). Here, TH34 treatment also dose-dependently increased γH2AX-positive cells after 24 h, albeit with a relatively small effect size (Fig. 5j–l). Treatment of NB8 cells with increasing doses of TH34 revealed a reduction of cellular metabolic activity at micromolar doses, with an IC50 of 47.3 µM (Fig. 5m), which is slightly higher than that of Kelly cells (39.1 µM, Fig. 3). In summary, TH34 induces DNA damage independent of caspase activation prior to triggering caspase-dependent programmed cell death in high-grade- and primary neuroblastoma cells.

## Discussion

As a consequence of the pivotal roles of HDACs in various diseases including cancer, disruption of their activity with broad-spectrum or subtype-specific inhibitors has garnered strong interest in preclinical research and drug discovery (Witt et al. 2009a). With the development of techniques such as X-ray-based crystal structure analysis and computational approaches, a variety of novel selective HDAC inhibitors have emerged for laboratory and clinical application. We have previously identified high HDAC8 and HDAC10 expression to correlate with poor outcomes in neuroblastoma and high-grade neuroblastoma, respectively. In line with this, treatment of high-grade neuroblastoma cells with the selective HDAC8 inhibitor PCI-34051 induces cellular differentiation (Rettig et al. 2015) and the class IIb HDAC inhibitor tubastatin A interferes with lysosomal trafficking and cellular stress-response mechanisms such as autophagy, rendering tumor cells more susceptible to cytotoxic treatment (Oehme et al. 2013). We thus aimed to develop an HDAC inhibitor with a target spectrum covering HDACs 8 and 10 and sparing HDACs 1, 2 and 3, as dose-limiting side effects of clinically used HDAC inhibitors are attributed to the inhibition of these HDAC subtypes (Witt et al. 2009b).

Here, we describe the newly developed HDAC6/8/10 inhibitor TH34, identified in a screen, in which novel HDAC inhibitory compounds were evaluated for their on- and off-target activities via phenotypical assays and analysis of protein acetylation patterns. TH34 treatment induced hyperacetylation of tubulin and SMC3, established bona fide substrates of HDAC6 and 8, respectively, indicating inhibition of these HDAC subtypes (Deardorff et al. 2012; Decroos et al. 2015). Although even more candidate HDAC8 substrates were recently identified via genetically encoded active site photo-crosslinking (Lopez et al. 2017), the identification of HDAC10-specific substrates remains difficult. To date, no antibody against a bona fide-acetylated HDAC10 substrate has been described. Accumulation of acidic vesicular organelles has been shown to be directly linked to HDAC10 inhibition and quantification of this phenotype can be used to measure cellular HDAC10 activity. As such, TH34 treatment induced strong accumulation of acidic vesicular organelles.

Throughout the cell cycle, SMC3 undergoes a cycle of acetylation and deacetylation, which is essential for the functions of the cohesin complex consisting of SMC1, RAD21, STAG1/2 and SMC3. This complex mediates sister chromatid cohesion during mitosis by forming a connecting ring in S phase, which disintegrates during mitotic anaphase, guaranteeing equal distribution of chromatids (Beckouet et al. 2010). As recent studies suggest that SMC3 hyperacetylation inhibits sister chromatid release at anaphase (Beckouet et al. 2016; Gligoris et al. 2014), it is conceivable that the HDAC8 inhibitory function of TH34 is responsible for the increase of mitotic aberrations and G2/M cell cycle arrest in neuroblastoma cells after treatment with the novel HDAC6/8/10 inhibitor.

Recent advances in HDAC10 research include the identification of its crystal structure, which provided evidence that HDAC10 might be a polyamine rather than a lysine deacetylase (Hai et al. 2017). In neuroblastoma, the polyamine regulating *ODC1* was found co-upregulated with *MYCN* and correlates with poor outcome in neuroblastoma, suggesting a detrimental role in neuroblastoma biology (Gamble et al. 2012; Hogarty et al. 2008; Lastowska et al. 2007). Moreover, polyamines are known to modulate DNA conformation by strongly binding to the DNA helix (Feuerstein et al. 1990; Matthews 1993), and recently identified roles of HDAC10 include regulation of DNA mismatch and DSB repair in various cancer types such as ovarian carcinoma (Islam et al. 2017; Radhakrishnan et al. 2015). DNA damage has also been described as a result of HDAC6 depletion and inhibition (Namdar et al. 2010; Wang et al. 2012), and the above-mentioned HDAC8-dependent cohesin complex is well known to accumulate at DNA break sites to mediate DNA repair and recruit cell cycle checkpoint-activating proteins (Caron et al. 2012; Watrin and Peters 2009). We thus hypothesized that combined HDAC6/8/10 inhibition affects DNA damage repair mechanisms. During the first 24 h of treatment with the HDAC6/8/10 inhibitor TH34, we detected a marked increase in γH2AX foci, providing strong evidence of the involvement of HDACs 6, 8 and 10 in DNA damage repair mechanisms in neuroblastoma. Importantly, this effect could not be reverted by co-treatment with the caspase inhibitor Z-VAD-FMK, indicating that DSB accumulation under TH34 treatment is a process independent of caspase activation.

Overall, these results qualify TH34 as a promising targeted agent for further development as a neuroblastoma therapeutic, including in vivo toxicity and efficacy testing. However, physiologically relevant aspects, not covered by our study, such as metabolic half life and immunosuppression by HDAC inhibitors, especially class IIb HDAC inhibitors (Kalin et al. 2012), which might limit applicability and anti-tumoral efficacy of TH34 in immunocompetent organisms, are mandatory to be evaluated in future studies. In conclusion, the selective HDAC6/8/10 inhibitor TH34 effectively and selectively eliminates high-grade neuroblastoma cells while sparing non-transformed human cells. In neuroblastoma cell lines as well as primary neuroblastoma cells, it markedly induces DNA damage, followed by differentiation and G2/M phase cell cycle arrest at later timepoints, eventually leading to cell death. Besides its potential as a targeted therapeutic in high-grade neuroblastoma, TH34 also serves as a valuable tool compound in laboratory research for investigating the roles of histone deacetylases in health and disease.

## Electronic supplementary material

Below is the link to the electronic supplementary material.

Supplementary material 1 (DOCX 39 KB)

**Suppl. Fig. 1 TH34 displays no selectivity for HDAC3 and class IIa HDACs (a)** Dose–response curve of TH34 tested against recombinant HDAC3 using a fluorogenic p53 peptide. **(b)** Dose–response curve of TH34 tested against whole cell lysate using the selective class IIa HDAC-fluorogenic substrate. **(c)** Selectivity control using the class IIa HDAC inhibitor MAZ1866 against whole cell lysate with the selective class IIa fluorogenic substrate. **(a-c)** Mean values as well as SD are represented. (TIF 318 KB)

**Suppl. Fig. 2 TH34-induced cell death cannot be rescued by N-acetylcysteine, necrostatin-1 or trolox (a-c)** Proportion of dead SK-N-BE(2)-C cells after treatment with indicated concentrations of TH34 for 72 hours with or without N-acetylcysteine (NAC, 10 mM, **(a)**), necrostatin-1 (25 µM, **(b)**) or trolox (100 µM, **(c)**), determined via automated trypan blue staining. Bar graphs represent mean values of at least three independent experiments performed in triplicates and statistical analysis was performed using unpaired, two-tailed *t* test (***: p < 0.001; **: 0.001 ≤ p < 0.01; *: 0.01 ≤ p < 0.05, ns: not significant). Error bars represent SD. (TIF 418 KB)

**Suppl. Fig. 3 TH34 enhances retinoid-induced neuron-like differentiation and synergizes with ATRA to reduce colony growth capacity of SK-N-BE(2)-C neuroblastoma cells (a)** Phenotype of SK-N-BE(2)-C neuroblastoma cells treated with TH34 (10 µM) with or without ATRA (10 µM) for 6 days. Three independent experiments were performed in triplicate, and this figure shows results from one representative experiment. **(b)** Dose-dependent reduction of SK-N-BE(2)-C colony growth after treatment with indicated doses of TH34 and ATRA for 4 days and regrowth of colonies in fresh medium for 7 days. **(c)** SK-N-BE(2)-C colony growth (CG) after treatment with indicated concentrations of TH34 and ATRA for 4 days and regrowth of colonies in fresh medium for 7 days, normalized to solvent control and quantified using ImageJ version 1.49v. **(d)** Combination indices (CI) determined from quantified colony growth after combined treatment with low concentrations of TH34 and ATRA, indicating synergism. Analysis was performed using the CompuSyn synergism calculation software based on the Chou–Talalay method (Chou 2010). (TIF 5374 KB)

**Fig. 4 TH34 increases nuclear size as well as abundance of aberrant mitotic figures**. Fluorescence microscopic analysis of nuclear size and morphology in SK-N-BE(2)-C cells treated with TH34 (10 µM) for six days. Presented are five replicates per condition. Nuclei were stained with DAPI. (TIF 5183 KB)
