# Supplementary material for: The HDAC6/8/10 inhibitor TH34 induces DNA damage-mediated cell death in human high-grade neuroblastoma cell lines
Source: Arch Toxicol. 2018 Jun 9;92(8):2649–64. doi: 10.1007/s00204-018-2234-8 (PMC6063332; doi:10.1007/s00204-018-2234-8)
Supplement: Supplementary file 1 — Supplementary material 1 (DOCX 39 KB) [file 204_2018_2234_MOESM1_ESM.docx]

## Supplementary Table 1

| **target HDAC** | **class I** | | | **class IIa^3^** | **class IIb** |
| --- | --- | --- | --- | --- | --- |
|  | **HDAC1^1^** | **HDAC3^2^** | **HDAC8^1^** |  | **HDAC6^1^** |
| **biochemical IC50 [µM]** | 21.8 ± 2.1 | 78.3 ± 0.9 | 0.26 ± 0.04 | > 200 µM | 5.1 ± 0.3 |

**TH34 is selective for HDAC8 and HDAC6 over other class I and class IIa HDACs**

^1^ Fluorometrically determined biochemical IC50 values using recombinant human HDAC1, HDAC6 and HDAC8, as published previously ([Heimburg et al. 2017](#_ENREF_1)).

^2^ Biochemical HDAC3 IC50 value, determined using a fluorogenic p53 peptide, detailed results depicted in **Supplementary Figure 1 a**.

^3^ Result depicted in **Supplementary Figure 1 b**, using whole cell lysate with the selective class IIa HDAC fluorogenic substrate, indicating no relevant inhibition of class IIa HDACs at the highest tested dose (200 µM).
