# Supplementary figures and images for: The HDAC6/8/10 inhibitor TH34 induces DNA damage-mediated cell death in human high-grade neuroblastoma cell lines
Source: Arch Toxicol. 2018 Jun 9;92(8):2649–64. doi: 10.1007/s00204-018-2234-8 (PMC6063332; doi:10.1007/s00204-018-2234-8)

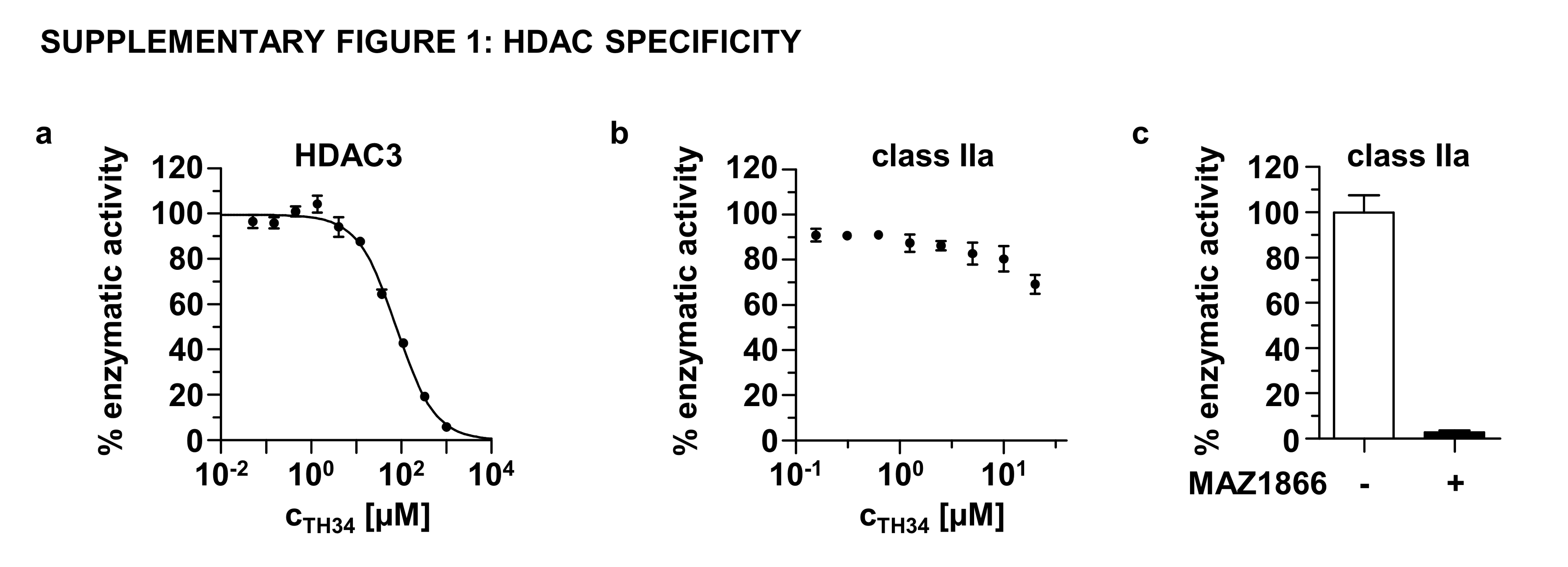

Supplement: Supplementary file 2 — Suppl. Fig. 1 TH34 displays no selectivity for HDAC3 and class IIa HDACs (a) Dose–response curve of TH34 tested against recombinant HDAC3 using a fluorogenic p53 peptide. (b) Dose–response curve of TH34 tested against whole cell lysate using the selective class IIa HDAC-fluorogenic substrate. (c) Selectivity control using the class IIa HDAC inhibitor MAZ1866 against whole cell lysate with the selective class IIa fluorogenic substrate. (a-c) Mean values as well as SD are represented. (TIF 318 KB) [file 204_2018_2234_MOESM2_ESM.tif]

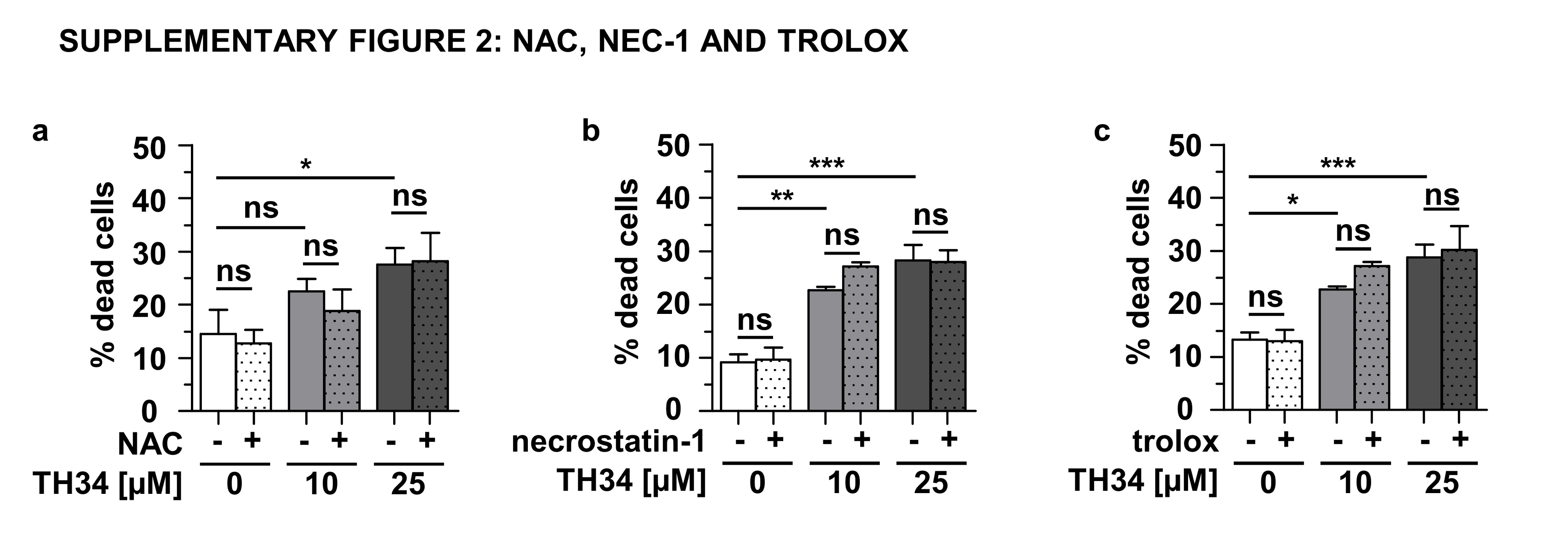

Supplement: Supplementary file 3 — Suppl. Fig. 2 TH34-induced cell death cannot be rescued by N-acetylcysteine, necrostatin-1 or trolox (a-c) Proportion of dead SK-N-BE(2)-C cells after treatment with indicated concentrations of TH34 for 72 hours with or without N-acetylcysteine (NAC, 10 mM, (a)), necrostatin-1 (25 µM, (b)) or trolox (100 µM, (c)), determined via automated trypan blue staining. Bar graphs represent mean values of at least three independent experiments performed in triplicates and statistical analysis was performed using unpaired, two-tailed t test (***: p < 0.001; **: 0.001 ≤ p < 0.01; *: 0.01 ≤ p < 0.05, ns: not significant). Error bars represent SD. (TIF 418 KB) [file 204_2018_2234_MOESM3_ESM.tif]

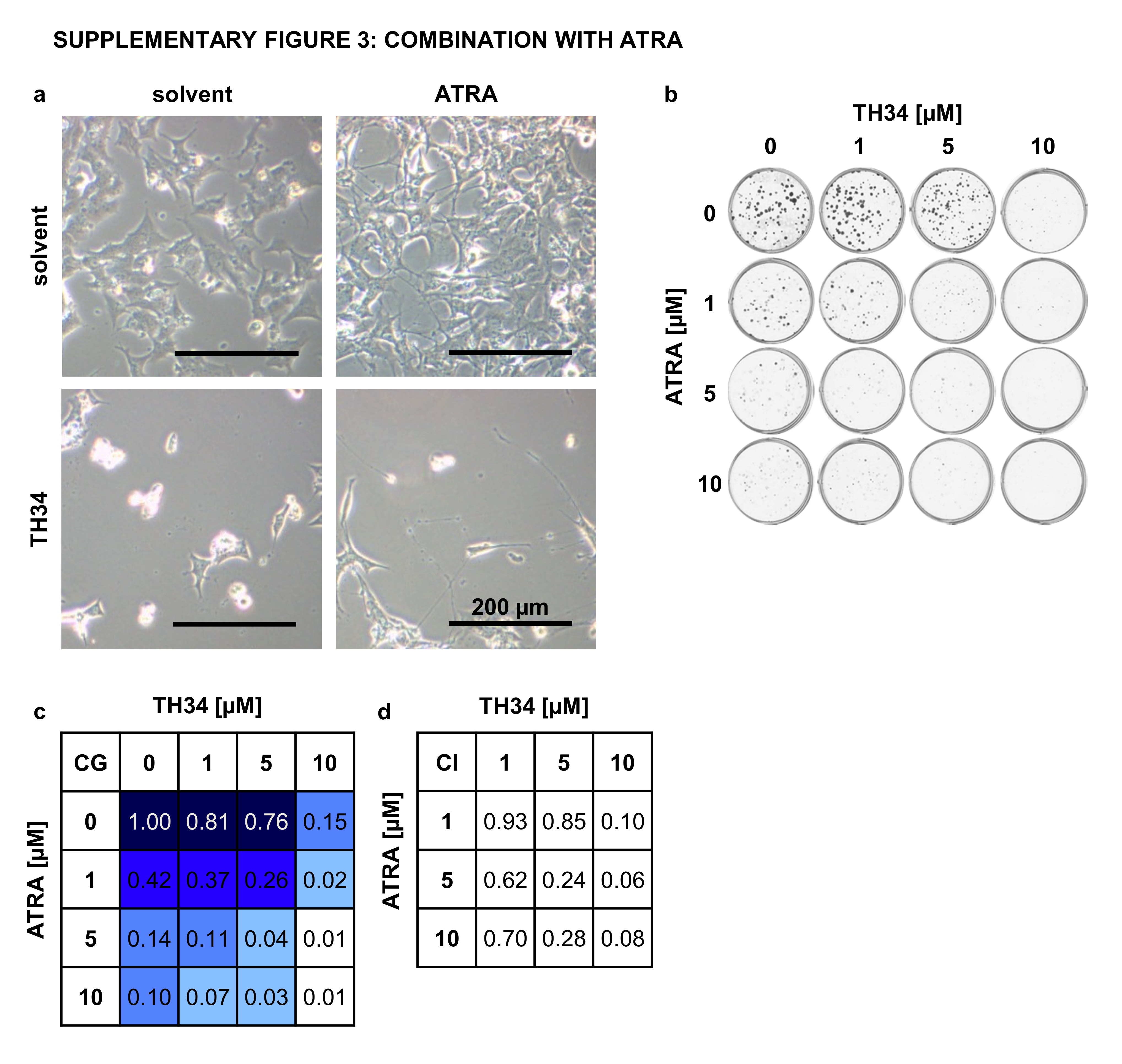

Supplement: Supplementary file 4 — Suppl. Fig. 3 TH34 enhances retinoid-induced neuron-like differentiation and synergizes with ATRA to reduce colony growth capacity of SK-N-BE(2)-C neuroblastoma cells (a) Phenotype of SK-N-BE(2)-C neuroblastoma cells treated with TH34 (10 µM) with or without ATRA (10 µM) for 6 days. Three independent experiments were performed in triplicate, and this figure shows results from one representative experiment. (b) Dose-dependent reduction of SK-N-BE(2)-C colony growth after treatment with indicated doses of TH34 and ATRA for 4 days and regrowth of colonies in fresh medium for 7 days. (c) SK-N-BE(2)-C colony growth (CG) after treatment with indicated concentrations of TH34 and ATRA for 4 days and regrowth of colonies in fresh medium for 7 days, normalized to solvent control and quantified using ImageJ version 1.49v. (d) Combination indices (CI) determined from quantified colony growth after combined treatment with low concentrations of TH34 and ATRA, indicating synergism. Analysis was performed using the CompuSyn synergism calculation software based on the Chou–Talalay method (Chou 2010). (TIF 5374 KB) [file 204_2018_2234_MOESM4_ESM.tif]

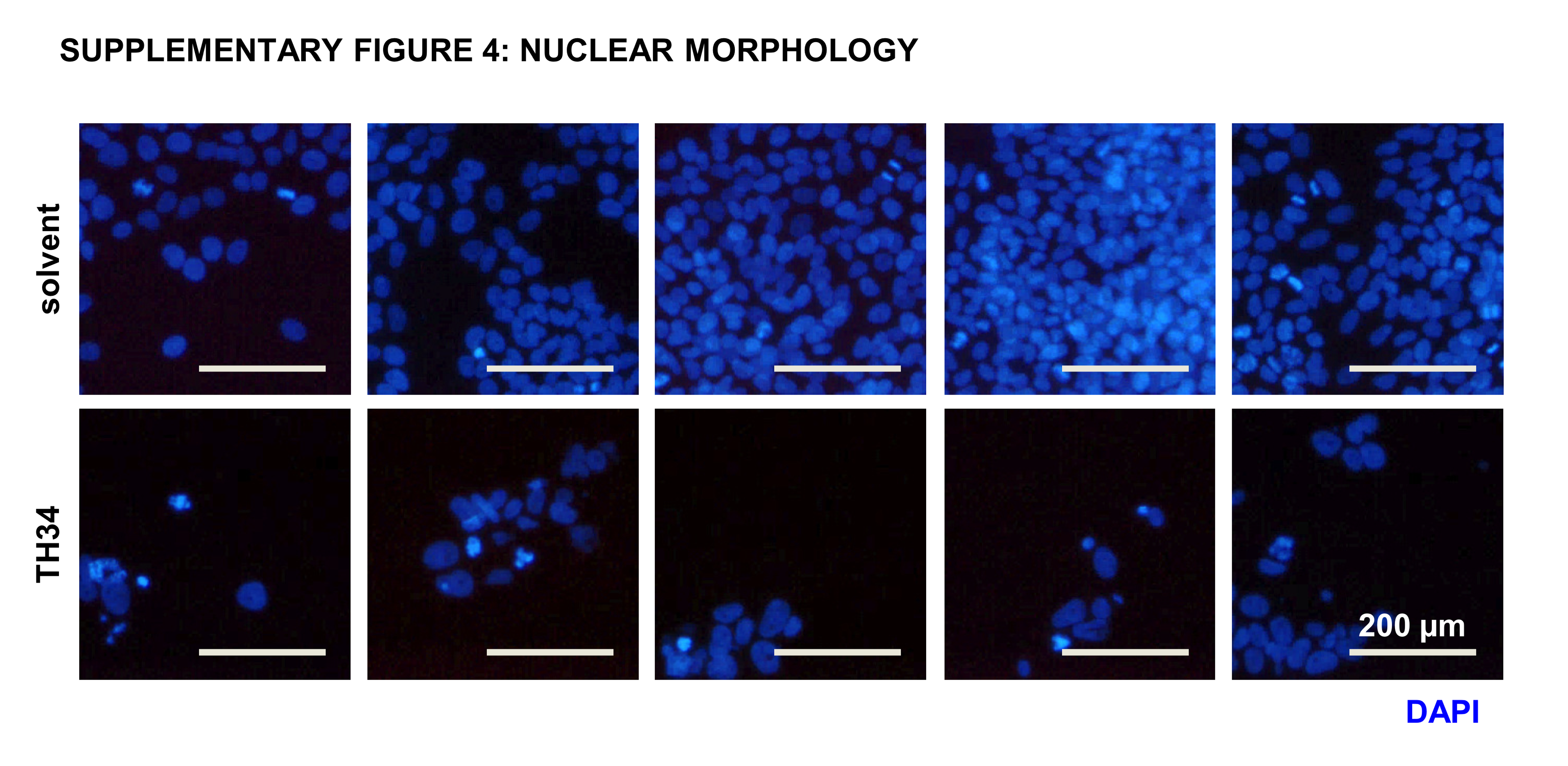

Supplement: Supplementary file 5 — Fig. 4 TH34 increases nuclear size as well as abundance of aberrant mitotic figures. Fluorescence microscopic analysis of nuclear size and morphology in SK-N-BE(2)-C cells treated with TH34 (10 µM) for six days. Presented are five replicates per condition. Nuclei were stained with DAPI. (TIF 5183 KB) [file 204_2018_2234_MOESM5_ESM.tif]
